# Structural and functional characterization of the PDZ domain of the human phosphatase PTPN3 and its interaction with the human papillomavirus E6 oncoprotein

**DOI:** 10.1038/s41598-019-43932-x

**Published:** 2019-05-15

**Authors:** Mariano Genera, Damien Samson, Bertrand Raynal, Ahmed Haouz, Bruno Baron, Catherine Simenel, Raphael Guerois, Nicolas Wolff, Célia Caillet-Saguy

**Affiliations:** 1 Récepteurs-Canaux, Institut Pasteur, UMR 3571, CNRS, F-75724 Paris, France; 2 Sorbonne Université, Complexité du Vivant, F-75005 Paris, France; 3 RMN des biomolécules, Institut Pasteur, UMR 3528, CNRS, F-75724 Paris, France; 4 Plate-forme de Biophysique Moléculaire, Institut Pasteur, UMR 3528, CNRS, F-75724 Paris, France; 5 Plate-forme de Cristallographie, Institut Pasteur UMR 3528, CNRS, F-75724 Paris, France; 6 Institut de Biologie Intégrative de la Cellule (I2BC), CEA, CNRS, Université Paris-Sud, Université Paris-Saclay, 91190 Gif-sur-Yvette, Cedex France

**Keywords:** Structural biology, Molecular conformation, NMR spectroscopy, X-ray crystallography

## Abstract

The human protein tyrosine phosphatase non-receptor type 3 (PTPN3) is a PDZ (PSD-95/Dlg/ZO-1) domain-containing phosphatase with a tumor-suppressive or a tumor-promoting role in many cancers. Interestingly, the high-risk genital human papillomavirus (HPV) types 16 and 18 target the PDZ domain of PTPN3. The presence of a PDZ binding motif (PBM) on E6 confers interaction with a number of different cellular PDZ domain-containing proteins and is a marker of high oncogenic potential. Here, we report the molecular basis of interaction between the PDZ domain of PTPN3 and the PBM of the HPV E6 protein. We combined biophysical, NMR and X-ray experiments to investigate the structural and functional properties of the PDZ domain of PTPN3. We showed that the C-terminal sequences from viral proteins encompassing a PBM interact with PTPN3-PDZ with similar affinities to the endogenous PTPN3 ligand MAP kinase p38γ. PBM binding stabilizes the PDZ domain of PTPN3. We solved the X-ray structure of the PDZ domain of PTPN3 in complex with the PBM of the HPV E6 protein. The crystal structure and the NMR chemical shift mapping of the PTPN3-PDZ/peptide complex allowed us to pinpoint the main structural determinants of recognition of the C-terminal sequence of the E6 protein and the long-range perturbations induced upon PBM binding.

## Introduction

Protein tyrosine phosphatases (PTPs) play critical roles in cell signaling pathways. They are known to regulate a variety of cellular processes including cell growth, differentiation, mitotic cycle, and oncogenic transformation. Together with kinases, they control the balance of phosphorylated species, enabling specific and varied signaling responses.

**Figure 1:**
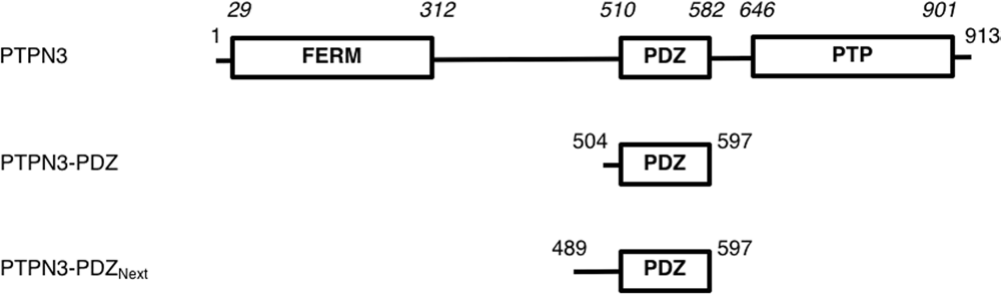
Schematic representation of the PTPN3 constructs. Numbers on both extremities indicate the boundary residues for each construct; Numbers in italic above the schematic construct of full-length PTPN3 correspond to the boundary residues of each protein domain from Uniprot P26045.

The human protein tyrosine phosphatase non-receptor type 3 (PTPN3) and the protein tyrosine phosphatase non-receptor type 4 (PTPN4) compose the NT5 type of non-transmembrane PTPs. These large modular proteins feature a N-terminal FERM (Band 4.1, Ezrin, Radixin, and Moesin) domain that is responsible for the localization of these enzymes to submembranous sites in the cell, a PDZ (PSD-95/Dlg/ZO-1) domain that allows specific binding to C-terminal recognition sequences in target proteins, and a C-terminal catalytic tyrosine phosphatase domain (Fig. 1). Three different isoforms of PTPN3 are known to be produced by alternative splicing, one being the full-length protein (UniProt P26045-1), whereas the FERM domain is partially or totally missing in the other two (UniProt P26045-2 and P26045-3). PTPN3 is localized in the cytoplasm and enriched at the plasma membrane, although in absence of the FERM domain it is exclusively cytoplasmic^1^.

PTPN3 has been linked to many cancers either with a tumor-suppressive or a tumor-promoting role^2^. Interestingly, PTPN3 is targeted by oncoviruses such as the high-risk genital human papillomaviruses (HPV) types 16 and 18^3^ and the hepatitis B virus (HBV)^4^. HPV types 16 (HPV16) and 18 (HPV18) are responsible of 70% of cervical cancers and precancerous cervical lesions^5^.

The E6 proteins of HPV16 and HPV18 possess a conserved C-terminal PDZ binding motif (PBM), which mediates interaction with some cellular PDZ domain-containing proteins. The ability of E6 to bind to PDZ domain-containing proteins correlates with its oncogenic potential^6^. The PDZ domain of PTPN3 (PTPN3-PDZ) has been identified as a target of the E6 PBM of HPV16 and 18. This PDZ-PBM interaction results in the ubiquitin-mediated proteasomal degradation of PTPN3. Therefore, the endogenous levels of PTPN3 are particularly low in HPV-positive cervical carcinoma cell lines^3^.

To date, the structure of PTPN3-PDZ is not known and the PDZ-mediated interactions between PTPN3 and viral partners have not been described. Indeed, the few biophysical and structural studies on PTPN3 have focused on the PTP domain in complex with phospho-peptide substrates derived either from the mitogen-activated protein kinase (MAPK) p38γ (also known as MAPK12)^7^ or the Epidermal growth factor receptor substrate 15 (Eps15)^8^. We have previously shown that the PDZ domain of the closely related PTPN4 inhibits its phosphatase activity^9,10^. Similarly, the inhibitory role of the PDZ domain of PTPN3 on the catalytic activity has been reported using a phospho-p38γ peptide as a substrate for kinetics experiments^7^.

In this study, we report the structural and functional analyses of the PDZ domain of PTPN3. We investigated the molecular mechanism of interaction between the PDZ domain of PTPN3 and the PBM of cellular and viral partners (Table 1), the MAP kinase p38γ, the HBV core protein and the HPV E6 protein. We employed an integrative approach based on biophysical, nuclear magnetic resonance (NMR) and X-ray crystallography experiments. We showed that the C-terminal PBM sequences of the HPV E6 protein interact with PTPN3-PDZ with similar affinities to those of the endogenous PTPN3 ligand p38γ and the HBV Core protein^4^. By solving the X-ray structure and mapping the NMR chemical shift changes of PTPN3-PDZ in complex with the PBM of HPV16 E6, we identified the molecular basis of recognition of the C-terminal sequence of E6 protein and the induced perturbations that spread from the binding groove.

**Table 1:** Peptide sequences, affinities for PTPN3-PDZ and melting temperature of complexes.

Peptides	Sequences	K_D_ (μM)	Tm (°C)
p38γ PBM	SWARVSKETPL	26 ± 25	45
HBVc PBM	RRRRSQSRESQC	29 ± 24	47
HPV16E6 PBM	RSSRTRRETQL	53 ± 31	52
HPV18E6 PBM	RQERLQRRETQV	37 ± 20	49

The errors are the standard deviations of all the K_D_ values derived from 8 to 14 curves fitted for each titration.

## Results

### Quality control of the recombinant PTPN3-PDZ domain

Presently, there is no structural information on PTPN3-PDZ other than that provided by homology modelling^7^. To bridge this gap, we performed different structural and biophysical studies on the recombinant PTPN3-PDZ construct (Fig. 1) expressed in *Escherichia coli*.

**Figure 2:**
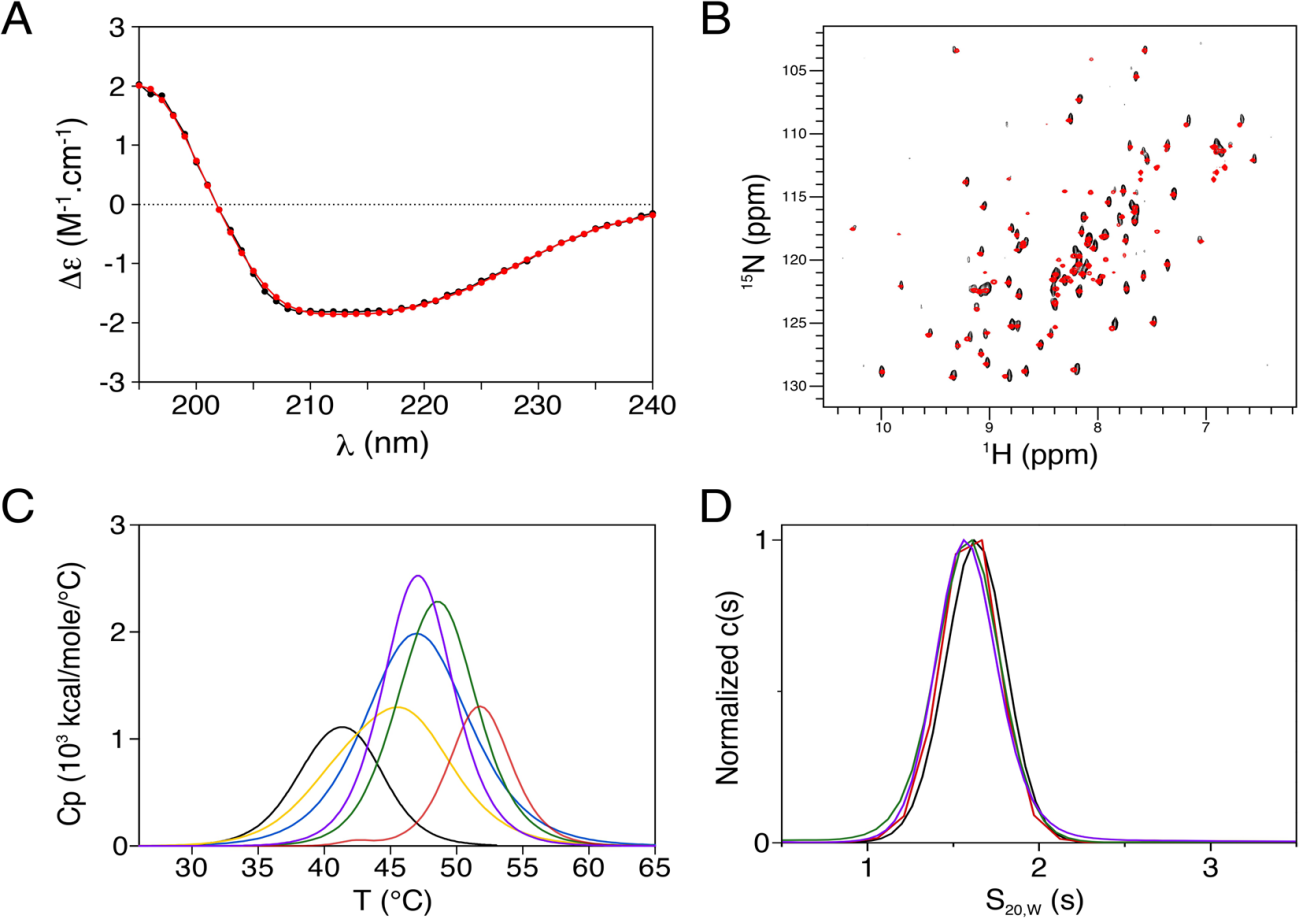
Stability, oligomerization and structural characterization of PTPN3-PDZ. (**A**) Far-UV CD spectrum (195–240 nm) of PTPN3-PDZ. The experimental spectrum of PTPN3-PDZ and the best fit obtained with the CONTINLL algorithm are represented as black and red lines, respectively. (**B**) Superimposed ^1^H, ^15^N HSQC spectra of PTPN3-PDZ (black) and PTPN3-PDZ_Next_ (red). (**C**) DSC thermograms of PTPN3-PDZ unbound and complexed to PBM peptides. Free PTPN3-PDZ curve is shown as a black line, and PTPN3-PDZ complexed to p38γ PBM, HBVc PBM and HPV16E6 PBM and HPV18E6 PBM curves are shown as yellow, purple, green and red lines, respectively. The DSC thermogram of free PTPN4-PDZ is colored in blue. (**D**) Sedimentation coefficient distributions of PTPN3-PDZ_Next_ unbound and complexed to viral PBM peptides. The black, red, green and purple lines correspond to PTPN3-PDZ_Next_ unbound and complexed to HPV18E6PBM, HPV16E6 PBM and HBVc PBM, respectively.

To estimate the secondary structure content of the PDZ construct, we performed circular dichroism (CD) measurements in the far-UV (195–240 nm). The PTPN3-PDZ spectrum (Fig. 2A) appeared to be of mixed α-helix and β-sheet content, which upon deconvolution indicated 16% α-helix and 32% β-sheet. The secondary structure content extracted from the X-ray structure of the PDZ domain of its close homologue PTPN4 (PTPN4-PDZ) (PDB id 3NFK)^11^ is 17% α-helix and 31% β-sheet. Thus, the PTPN3-PDZ construct presents a secondary structure content similar to that of PTPN4-PDZ.

We assessed the tertiary folding of PTPN3-PDZ by NMR by performing ^15^N heteronuclear single quantum correlation (HSQC) experiment. The high dispersion of resonances in the HSQC spectrum of PTPN3-PDZ (Fig. 2B) is indicative of a mainly well-folded construct in solution.

### Stability and oligomeric state of PTPN3-PDZ

To evaluate the stability of PTPN3-PDZ, we performed differential scanning calorimetry (DSC) (Fig. 2C). A single endothermic peak was observed in the DSC thermogram, with a thermal transition midpoint (T_m_) of 41 °C. As a comparison, we performed DSC on PTPN4-PDZ. We obtained an endothermic peak with a T_m_ of 47 °C (Fig. 2C). The data show that PTPN3-PDZ is less stable in solution than PTPN4-PDZ. This thermal instability of the PTPN3-PDZ construct could be responsible for its aggregation previously detected by NMR. Indeed, we observed a significant loss of peak intensities in the HSQC spectrum of PTPN3-PDZ related to aggregation after 10 h at 20 °C (data not shown).

To estimate the oligomeric state of the PTPN3-PDZ construct, analytical ultracentrifugation (AUC) experiments were performed (Table 2). Three different concentrations of the protein were tested, 10, 25 and 70 μM. Only one single peak was detected at 10 μM with a sedimentation coefficient of 1.5S in agreement with a monomeric form of 10.5 kDa. At 25 μM, an additional peak was detected with a sedimentation coefficient of 2.6S corresponding to a dimeric form. At a concentration of 70 μM, higher oligomeric species could be detected. Thus, the AUC data of the PTPN3-PDZ construct reveal a concentration-dependent equilibrium between monomer, dimer and higher oligomeric states with only monomers detected at a concentration of 10 μM.

**Table 2:** Hydrodynamic parameters of PTPN3-PDZ derived from the analysis of analytical ultracentrifugation.

PTPN3-PDZ						
	10 μM	25 μM	70 μM	70 μM + HBVc PBM		
MW theoretical (kDa)	10.5	10.5	10.5	12.0		
MW estimated (kDa)	12.4	11.3–25.9^a^	nc	13.5		
Sed coef (S_0,w,20_) (S)	1.5	1.5–2.6^a^	nc	1.6		
**PTPN3-PDZ**_**Next**_						
	**17**.**5** **μM**	**35** **μM**	**70** **μM**	**70** **μM** + **HPV16E6 PBM**	**70** **μM** + **HPV18E6 PBM**	**70** **μM** + **HBVc PBM**
MW theoretical (kDa)	12.7	12.7	12.7	14.1	14.1	14.2
MW estimated (kDa)	12.7	12.5	15	13.7	14.7	15.6
Sed coef (S_0,w,20_) (S)	1.6	1.7	1.6	1.6	1.6	1.6

^a^Two peaks are present; nc: not calculated because of a continuum of species.

### PBM-mediated stabilization of the PTPN3-PDZ

To check whether the PBM binding to the PDZ domain could affect the stability of PTPN3-PDZ, we performed DSC and AUC on PTPN3-PDZ in complex with the p38γ PBM and the viral PBMs of HBV Core and HPV 16 and 18 E6 proteins, namely HBVc PBM, HPV16E6 PBM and HPV18E6 PBM respectively (Table 1). These PBMs have been shown to interact with the PTPN3-PDZ^3,4,7^. A single endothermic peak was observed in the DSC thermograms of all complexes (Fig. 2C) with a Tm of 45 °C, 47 °C, 52 °C and 49 °C for PTPN3-PDZ complexed to p38γ PBM, HBVc PBM, HPV16E6 PBM and HPV18E6 PBM, respectively (Table 1). For all the PBMs tested, an increase of 4 to 11 °C in the Tm was observed, showing that the PBM binding onto PTPN3-PDZ stabilizes the domain, whether the PBM is of cellular or viral origin.

In addition, AUC experiments of the PTPN3-PDZ construct at 70 μM complexed to HBVc PBM show only one species with a sedimentation coefficient of 1.6S corresponding to a one-to-one complex with the peptide, without higher oligomeric species (Table 2). Thus, the PBM binding onto PTPN3-PDZ prevents oligomerization at high concentration.

### Affinities of the viral and the cellular PBMs for PTPN3-PDZ

The affinities of HPV16E6 PBM, HPV18E6 PBM, p38γ PBM and HBVc PBM for PTPN3-PDZ were measured by NMR titration. We followed ^1^H, ^15^N chemical shift perturbations of PTPN3-PDZ signals in the ^1^H-^15^N HSQC spectra as a function of increasing concentrations of the PBM peptide to determine the dissociation constant (K_D_) (Fig. S1). The p38γ PBM peptide binds to PTPN3-PDZ with a K_D_ value of 26 μM (Table 1). This affinity is 16-fold lower than the one previously reported for PTPN4-PDZ (K_D_ of 1.6 μM)^10^. We obtained K_D_ values of 29 μM, 53 μM and 37 μM for PTPN3-PDZ with HBVc PBM, HPV16E6 PBM and HPV18E6 PBM, respectively (Table 1). The K_D_ values are all in the same tenth-of-micromolar range for the viral PBMs and are close to the one of the cellular partner p38γ. The measured affinities fall in the standard 0.1–100 μM range^12^ for PDZ-PBM interactions. To rule out the possibility of an effect of self-association of PTPN3-PDZ on the K_D_ values, we compared the K_D_ values with the HPV16E6 and HPV18E6 PBM peptides fitted from the NMR data with the ones estimated from the binding intensities (BIs) obtained for the HPV16E6 and HPV18E6 PBM peptides measured from a quantitative screening assay against the human PDZome library, in which each PDZ domain is at a concentration of 4 μM and in excess of PBM peptides^12^. The BIs are directly related to the Kd. For PTPN3-PDZ, the estimated K_D_ values are 46 and 96 μM for the HPV16E6 and HPV18E6 PBMs respectively, in good agreement with our experimental results.

### Stabilization of PTPN3-PDZ for structural studies

The instability and tendency to oligomerize of PTPN3-PDZ made this construct unsuitable for crystallogenesis. To gain in stability and protein expression yield, we extended the N-terminal extremity of PTPN3-PDZ by 15-residues with its wild-type upstream sequence (PTPN3-PDZ_Next_ in Fig. 1). These extensions have been reported to provide structural stability to some PDZ containing proteins^13,14^. The PTPN3-PDZ production yield was increased two-fold after extension from 0.9 mg to 1.8 mg of purified protein per liter of culture. The stability of PTPN3-PDZ_Next_ was then evaluated by NMR HSQC spectra recorded during a week at 20 °C. Spectra show a similar pattern of resonances than in the HSQC spectrum of PTPN3-PDZ without any signs of aggregation, such as a loss of peak intensity (Fig. 2B). The additional peaks of the N-terminal extension residues fall in the spectral region characteristic of unfolded protein and no chemical shift differences between PTPN3-PDZ_Next_ and the shorter form are detected. Thus, the N-terminal extension is not structured in the PTPN3-PDZ_Next_ and does not interact with the PDZ domain.

In addition, one single peak was detected by AUC for PTPN3-PDZ_Next_ at concentrations between 17.5 μM and 70 μM with a sedimentation coefficient of 1.6–1.7S, in agreement with a monomeric form of 12.7 kDa (Table 2) and devoid of higher oligomeric species. All these data indicate that the extension of PTPN3-PDZ resulted in a stable and folded PDZ domain in solution.

AUC experiments were also performed on the PTPN3-PDZ_Next_ construct at 70 μM complexed to HBVc PBM, HPV16E6 PBM and HPV18E6 PBM (Table 2 and Fig. 2D). As expected, only one species with a sedimentation coefficient of 1.6S is detected in all cases, corresponding to a one-to-one complex.

### Crystal structure of the PDZ domain of PTPN3 in complex with the viral HPV16E6 PBM

To investigate the molecular determinants of the interaction of PTPN3 with the HPV16 E6 protein, we solved the crystal structure of the complex formed by PTPN3-PDZ_Next_ and HPV16E6 PBM by molecular replacement at 2.19 Å resolution (Table 3). The structure factors and coordinates have been deposited in the Protein data Bank under accession code 6HKS.

**Table 3:** Data collection and refinement statistics.

	PTPN3-PDZ_Next_/HPV16E6 PBM
**Data collection**	
Space group	P 1 21 1
Unit cell (a, b, c) (Å)	46.62, 77.43, 130.03
α, β, γ (°)	90.00, 90.14, 90.00
Resolution (Å)	46.62–2.19 (2.23–2.19)
No. of reflections (total/unique)	324034/47024
Redundancy	6.9 (6.5)
Completeness (%)	99.1 (95.7)
*I*/σ(*I*)	8.17 (1.11)
*R-*meas¶	0.14 (1.71)
CC(1/2)	99.6 (72.6)
**Refinement**	
Resolution (Å)	43.34–2.19
No. reflections	46869
*R*_work_†/*R*_free_‡	0.195/0.245
No. of protein atoms/ligand atoms	4402/350
No. of solvent/hetero-atoms	235/9
Rmsd bond lengths (Å)	0.008
Rmsd bond angles (°)	0.949
*Wilson B*-factors	47.6
Ramachandran plot (favored/disallowed)*	97.4/0.4
PDB code	6HKS

Values in parenthesis correspond to the highest resolution shell.

^¶^Rmeas = Σh(n/n − 1)^1/2^Σi |Ii(*h*) − 〈I(h)〉|/ΣhΣi Ii(*h*), where Ii(*h*) and 〈I(*h*)〉 are t*h*e ith and the mean measurement, respectively, of the intensity of reflection h.

^†^Rwork = Σh||Fobs(h)| − |Fcalc(h)||/Σh|Fobs^(^h)|, where Fobs(h) and Fcalc(h) are the observed and calculated structure factors, respectively. No I/σ cutoff was applied.

^‡^Rfree is the R value obtained for a test set of reflections consisting of a randomly selected 5% subset of the data set excluded from refinement.

^*^Categories were defined by MolProbity.

**Figure 3:**
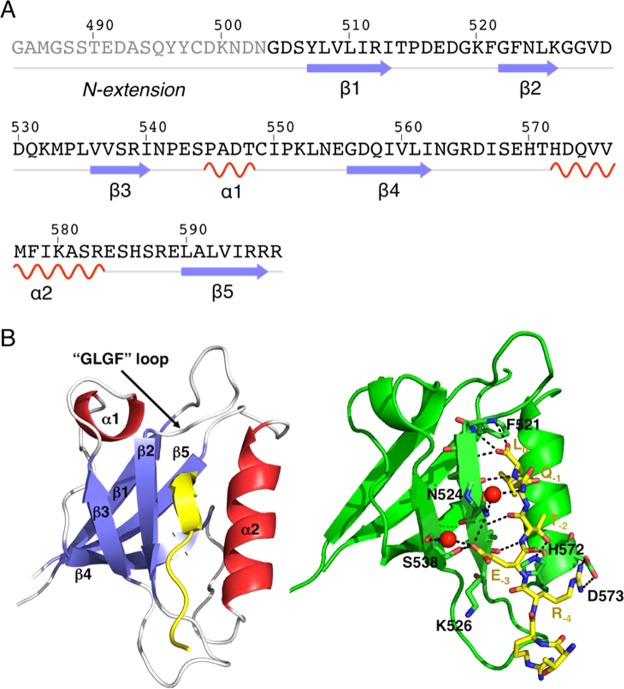
X-ray structure of PTPN3-PDZ_Next_ bound to HPV16E6 PBM. (**A**) Sequence and secondary-structure elements in PTPN3-PDZ_Next_. No electron density was observed for the residues shown in gray. (**B**) Left panel: Overall structure of PTPN3-PDZ_Next_ bound to HPV16E6 PBM displayed as a ribbon. The structure is colored according to secondary structure elements (β-strands in blue, helices in red, and loops in white). The peptide (in yellow) enters the PDZ-binding pocket delimited by the β2 strand, the α2 helix, and the “GLGF” loop ^519^GKFGF^523^. Right panel: Detailed view of the PTPN3-PDZ bound to HPV16E6 PBM. Important residues are shown as sticks in CPK colors, molecules of water are represented as red spheres, and intermolecular H-bonds and polar contacts are shown as black dashed lines.

PTPN3-PDZ_Next_ adopts a typical PDZ fold, with a β-sandwich comprising five β strands and two α helices (Fig. 3A). The PBM ligand binds in a hydrophobic cleft formed by the β2-strand, the α2-helix and the “GLGF” loop (Figs 3A,B). HPV16E6 PBM binds to the PDZ domain as an anti-parallel extension of the β2-strand domain in a conventional mode. There is no electron density corresponding to the N-terminal extension of PTPN3-PDZ_Next_ in the crystal, which indicates a disordered region, in agreement with the NMR results that show an unfolded extension.

**Figure 4:**
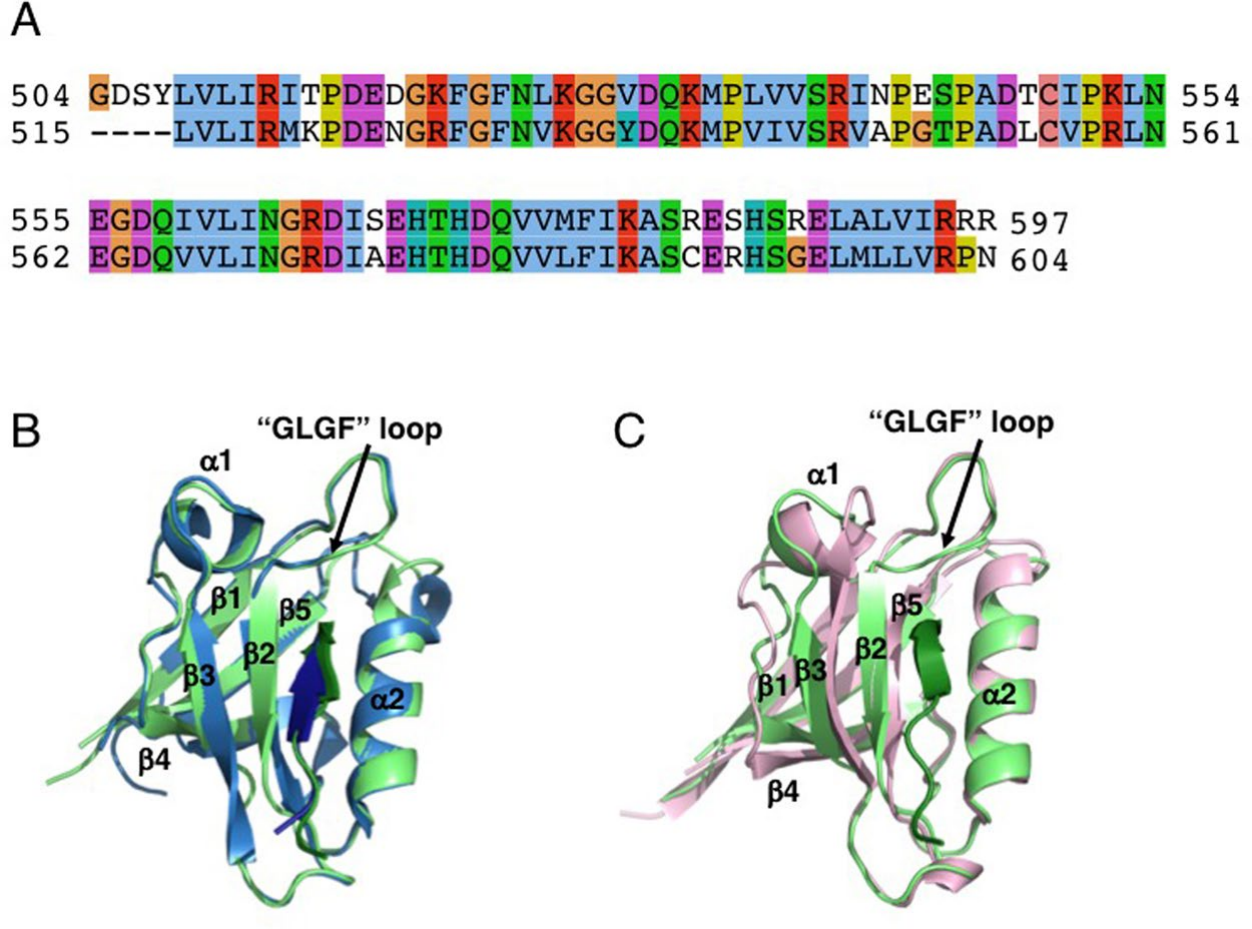
Structural comparison of the X-ray structure of PTPN3-PDZ_Next_ bound to HPV16E6 PBM with PTPN4-PDZ complexed to Cyto8retev, and with the CS-ROSETTA model of PTPN3-PDZ. (**A**) Clustalw2 alignment of PTPN3-PDZ (top) and PTPN4-PDZ (bottom). (**B**) Superposition of the structures of PTPN3-PDZ_Next_ bound to HPV16E6 PBM (light green/green) with PTPN4-PDZ bound to Cyto8retev (PDB 5EYZ) (light blue/blue). (**C**) Superposition of PTPN3-PDZ_Next_ bound to HPV16E6 PBM (light green/green) with the CS-ROSETTA model of PTPN3-PDZ (pink).

We also determined a 3D structural model with the CS-ROSETTA approach^15^ using the ^15^N, ^13^C_α_, ^13^CO NMR backbone and ^13^C_β_ resonances of PTPN3-PDZ_Next_ complexed to HPV16E6 PBM (BMRB accession number 27645). The CS-Rosetta modeling using solution NMR data revealed a very similar conformation to the crystal structure, with a low root mean square deviation (rmsd) of 1.11 Å for the backbone atoms between the crystal structure and the averaged solution model originated from the 10 lowest energy models (Fig. 4C). Thus, 3D models of PDZ-PTPN3 determined by CS-Rosetta reveal a conformation in solution that matches the crystal structure of the PDZ domain of the complex PTPN3-PDZ_Next_ and HPV16E6 PBM (Fig. 3B). The AUC sedimentation velocity was also back-calculated using the crystal structure^16^. We obtained a calculated sedimentation coefficient value of 1.59S, which is in good agreement with the experimental measurement of 1.6S. Thus, the overall structure of the domain in the crystal is consistent with its arrangement observed in solution.

Then, we compared the PDZ domains of PTPN3 and PTPN4, which share 71% of sequence identity (Fig. 4A). Both structures of PTPN3-PDZ and PTPN4-PDZ are highly similar, with a very low rmsd value of 0.34 Å for the backbone atoms (PTPN4 PDB ID 5EYZ)^10^ (Fig. 4B).

### Structural insights on the recognition of the PBM of HPV16 E6 protein

The PDZ domain of PTPN3 possesses the interaction network specific to class I PDZ domains and recognizes the consensus sequence S/T-X-Φ_COOH_, where X is any residue, and Φ is a hydrophobic residue^17^. A clear electron density map was seen for only the last seven C-terminal residues of HPV16E6 PBM (-TRRETQL_COOH_) that are inserted into the binding groove (Fig. 3A,B). Similarly, only the last 5 or 6 C-terminal residues of PBM peptides are visible in the crystal PTPN4-PDZ structures in complex with PBM peptides (PDB IDs 3NFL, 3NFK, 5EZO, 5EYZ in references^10,11^).

The interactions of the PBM residues at positions 0 and −2 with PTPN3-PDZ are quite similar to the bonding patterns already observed in the complex between PDZ-PTPN4 and the viral peptide Cyto13-att (-GETRL_-COOH_) derived from the attenuated rabies virus, or the optimized pro-apoptotic 13-amino acids peptide Cyto8-RETEV (-RETEV_-COOH_) (PDB IDs 3NFK and 5EYZ respectively)^10,11^. Indeed, the C-terminal carboxylate of the leucine (L_0_) of the HPV16E6 PBM forms three H-bonds with the amide nitrogens of F521, G522 and F523 of the “GLGF motif” on PTPN3-PDZ (Fig. 3B) as observed for F528, G529 and F530 of PTPN4-PDZ with the L_0_ of the Cyto13-att (-GETRL_-COOH_) PBM^11^. The hydroxyl group of threonine at position −2 forms a hydrogen bond with the Nε2 of the conserved histidine H572 from the α2-helix of PTPN3-PDZ (Fig. 3B). Electron acceptors such as serine and threonine are therefore preferred at this position for the class I PDZ domains.

At position −1 of HPV16E6 PBM, the glutamine (Q_−1_) side chain well defined and forms a H-bond with a water molecule that is also H-bonded to the Nδ2 of N524 of PTPN3-PDZ (Fig. 3B), whereas it is exposed to the solvent. Interestingly, in the complex of PTPN4-PDZ with the PBM of the glutamate receptor subunit GluN2A (PDB ID 3NFL), which presents a D in position −1^11^, the D_−1_ forms a H-bond with the N531 side-chain amine group of PTPN4-PDZ, the equivalent of N524 of PTPN3-PDZ.

A glutamate at position −3 (E_−3_) is conserved in all viral PBMs targeting PTPN3 and is also present in the one of p38γ. The side-chain carboxyl of E_−3_ forms a bifurcated H-bond with the hydroxyl of the conserved S538 (S545 for PTPN4) and N524 (N531 for PTPN4) amine group (Figs 3B and 4A). E_−3_ is also stabilized by hydrophobic contacts involving its Cβ-Cγ carbon chain and the long aliphatic side chain of K526 as observed in PTPN4 (K533) bound to Cyto8retev (PDB 5EYZ).

Finally, the guanidinium nitrogens of arginine at position −4 (R_−4_) form ionic bonds with the carboxylate oxygens of D573 (D580 in PTPN4)(Fig. 3B).

**Figure 5:**
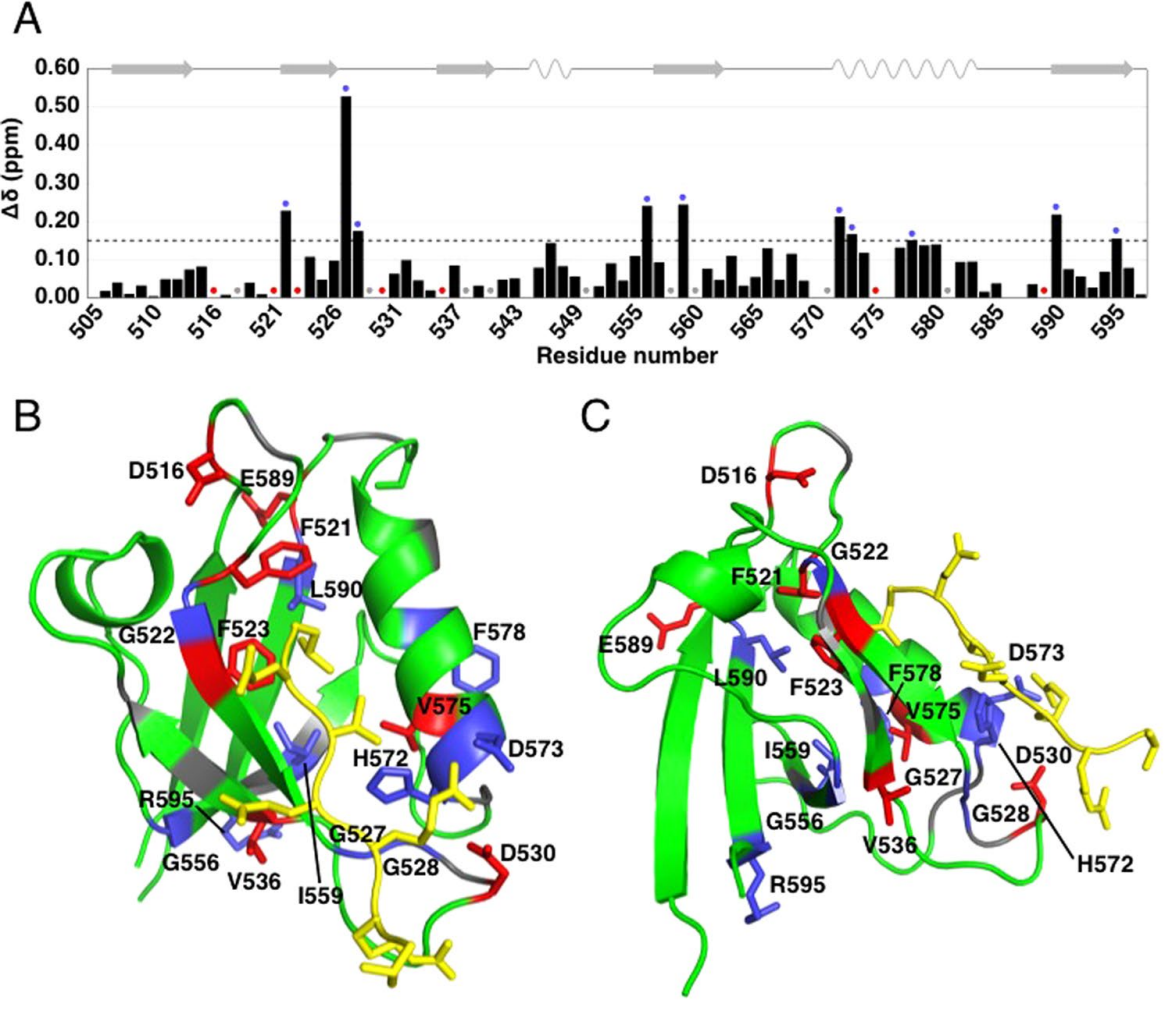
Δδ between free PTPN3-PDZ and PTPN3-PDZ bound to HPV16E6 PBM. (**A**) Δδ values (^1^H, ^15^N) computed as ∆δ = [(∆δH)^2^ + (∆δN*0.15)^2^]^1/2^. The dashed black line represents the threshold used to determine the perturbed region with ∆δ > 0.15 ppm; blue circles mark residues perturbed with ∆δ > 0.15 ppm; red circles mark residues with no resonance in free PTPN3-PDZ; grey circles mark residues whose behavior could not be safely defined, mainly because they fall in crowded spectral regions. (**B**) Two views of the structure of PTPN3-PDZ bound to HPV16E6 PBM (PDB ID 6HKS) with different colors depending on Δδ values. Blue: ∆δ > 0.15 ppm; red: resonances absent from the free PTPN3-PDZ spectrum; grey: residues whose resonances fall in crowded spectral regions. HPV16E6 PBM peptide is represented in yellow. Side chains of affected residues are represented as sticks.

To determine short- and long-range perturbations in PTPN3-PDZ upon PBM binding, we analyzed by solution NMR the chemical shifts of the free PTPN3-PDZ in solution and its complex with peptide HPV16E6 PBM, comparing their ^1^H, ^15^N HSQC spectra. We assigned 97% of the HN resonances, 99% of the Cα resonances, 90% of the Cβ resonances and 89% of the CO resonances (89 non-Proline residues over 94) of PTPN3-PDZ complexed to HPV16E6 PBM (BMRB accession number 27645). Chemical shift changes (Δδ) in PTPN3-PDZ spectra upon complex formation provide insights at atomic level on the residues involved in the interaction with the HPV16E6 PBM and on potential distal effects. Two types of signals were useful for the analysis of the ^1^H, ^15^N chemical shift mapping (Fig. 5A): (1) signals experiencing significant chemical shift changes (Δδ > 0.15 ppm); (2) signals that undoubtedly disappear from their original well-resolved position in the spectrum upon complex formation and are therefore severely affected when binding to the PBM (either due to large chemical shift changes or to severe line broadening effects caused by exchange). Residues corresponding to type 1 are shown in blue in Fig. 5A,B, while seven residues of type 2 are colored in red. Nine residues, whose behavior could not be safely defined mainly because they fall in crowded spectral regions, are colored in gray.

As expected, we identified differences in backbone NH chemical shifts between free PTPN3-PDZ and when bound to HPV16E6 PBM. Residues G522, G527 and H572, are among the most affected and are located in the PBM binding site. I559 and L590 also experience a strong effect. Indeed, they make hydrophobic contacts of 5.4 Å and 4 Å with F523 and F521 of the “GLGF motif”, respectively. E589 (in red), the direct neighbor of L590, displays perturbations both in chemical shift or intensity. Some residues of α2-helix in the binding site are affected: D573 and F578 (in blue) and V575 (in red). We find also residues such as F521 and F523 of the “GLGF” motif and D516 in the “GLGF” loop affected in their NMR resonances. D530 in the β2-β3 loop forms a H-bond with H572, which interacts directly with the PBM peptide (position T_−2_).

Interestingly, G556 (in red) is part of a path of perturbation starting from G527 (in red) and G528 (in blue) at the end of β2-strand in the binding groove, transmitted to the close V536 (in red) at the beginning of β3-strand, affected in signal intensity. The perturbation of V536 seems to be transferred to the close G556 on the β4-strand. I559 (in red) on β4-strand is also affected, possibly experiencing the perturbation of the F523 located 5 Å away of the “GLGF motif”. Finally, the path of perturbation finishes with R595 at the C-terminus of the PDZ domain (in blue). Thus, long-range perturbations seem to be induced upon PBM binding and transmitted through an interconnected network of residues throughout PTPN3-PDZ. This network involves the β2, β3 and β4 strands and the C-terminus.

## Discussion

PTPN3 is a PDZ domain-containing phosphatase that has been demonstrated to function as a tumor suppressor or, conversely, as an oncoprotein in a context-dependent manner. It integrates a number of signaling pathways via interactions mediated by its PDZ domain^18–20^. The PDZ domain of PTPN3 is also a cellular target of oncoviruses. Until now, no structural data on this domain were available. We investigated the structural and functional properties of PTPN3-PDZ and characterized its interaction with the PBMs of one cellular partner, the MAP kinase p38γ, and of its viral partners, the HPV E6 protein and the HBV core protein. We defined in particular the molecular basis of the PDZ-PBM interaction between HPV E6 and PTPN3.

### Structural properties of the PDZ domain of PTPN3

The crystal structure of PTPN3-PDZ_Next_ complexed to HPV16E6 PBM reveals a typical PDZ fold with five β-strands and two α-helices (Fig. 3A,B). It was previously reported that some PDZ domains can be stabilized by extensions of either the N-, C-, or both termini of a PDZ^21^. These extended regions were required for correct folding and ligand binding. For example, the PDZ1 of the membrane-associated guanylate kinase inverted MAGI-1 is unstable without N- and C-terminal extensions of 14 and 26 residues, respectively, even though these sequences are unstructured in both free and ligand-bound states^22^. Our minimal PTPN3-PDZ construct, deduced from sequence alignments with PDZ domains of known structure (Fig. 1), started at residue 504, while the first N-terminal secondary structure element observed in the crystal structure of PTPN3-PDZ_Next_, the β1-strand, started at residue 507 and not at 510 as expected from the sequence alignments. We showed that this PTPN3-PDZ construct is folded but unstable. We were able to increase its stability and production yield, while decreasing its tendency to auto-associate, by the addition of an unfolded 15-residue N-terminal extension to the minimal delimitation of PTPN3-PDZ. 3D structural models of PDZ-PTPN3 in complex with HPV16E6 PBM determined by CS-Rosetta using backbone and ^13^C_β_ NMR chemical shift assignments revealed a conformation in solution congruent with the X-ray structure of the PDZ domain.

Our AUC data showed that PTPN3-PDZ auto-association is prevented by PBM binding, as already reported for other PDZ domains^23^. The molecular mechanism by which PBM binding interferes with PTPN3 PDZ auto-association remains unknown. Our NMR results on long-range perturbations upon PBM binding are consistent with a network involving the β2, β3 and β4 strands and the C-terminus residues throughout PTPN3-PDZ. This connected pathway communicates the binding event to regions that are distal to the binding cleft. These findings are compatible with previous data on PDZ domains suggesting that energetic pathways within PDZ domains may support allostery^24–27^. A regulation controlled by the equilibrium between PBM binding and dimerization is attractive since over 30% of PDZ domains are known to form dimers in solution^28^.

### The PDZ domain of PTPN3 as a target of viruses

We showed that the viral PBMs interact with PTPN3-PDZ with similar affinities to that of the endogenous PTPN3 ligand p38γ. Despite variations in the PBM sequences, all the affinities fall in the 25–55 μM range (Table 1), in agreement with affinities previously reported for HPV E6 PBM PDZ binders^12^. This result is consistent with the hypothesis of a competition between cellular PBM-containing partners and PBMs on viral proteins to bind PTPN3 and hijack signalling pathways in infected cells. E6 binding to PTPN3 should be favoured in HPV infected cells since E6 is highly expressed in HPV-derived cancer cells. Only the C-termini of E6 oncoproteins in high-risk HPV strains contain PBMs, and this is a marker of high oncogenic potential. The presence of a PBM on E6 confers the capacity to interact with various cellular PDZ domain-containing proteins including PTPN3^29^. Furthermore, this interaction causes degradation of most of its targets through the ubiquitin–proteasome pathway^30^. Therefore, PDZ-protein interactions with E6 PBM not only disrupt protein–protein interactions but also promote degradation of the PDZ-containing protein. Our results raise the question of how specificity is achieved in this interaction, and whether or not PTPN3 PDZ domain is able to discriminate its binding partners.

### Molecular basis and specificities of the interaction with the PDZ domain of PTPN3

**Figure 6:**
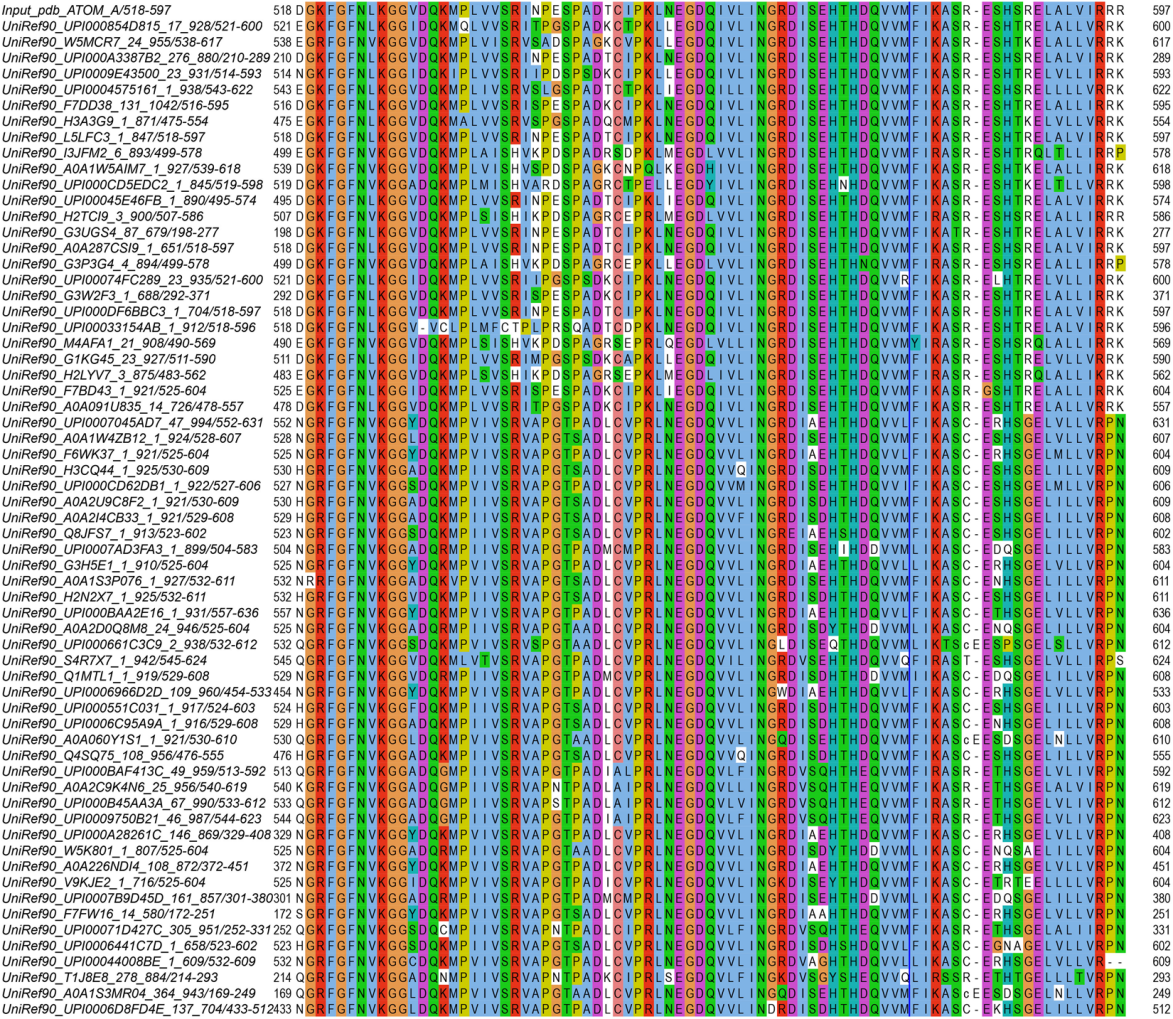
Sequence alignment of PTPN3 and PTPN4 from different species.

The crystal structure and the NMR chemical shift mapping of PDZ-PTPN3 in complex with the PBM of the E6 oncogenic protein of HPV16 highlight the main structural determinants of recognition of the C-terminal sequence of the E6 protein. HPV16E6 PBM binding to PTPN3-PDZ is consistent with the binding mode observed for canonical class I PDZ domains, where L_0_ and T_−2_ are involved in key interactions with the PDZ domain. Indeed, L_0_ interacts with the conserved GLGF motif, which is crucial for the hydrogen bond coordination of the terminal carboxylate group during the PDZ domain-PBM ligand interaction. Moreover, the T_−2_ forms a hydrogen bond with a conserved Histidine or Arginine in the PDZ domain, signature of class I PDZ domains^14,31^. The histidine, H572 in PTPN3, is very well conserved in the PTPN3 and PTPN4 orthologs (Fig. 6), and was also identified as a preferred residue for E6 binding by the alignment of 209 PDZ domains ranked according to their highest E6 binding intensity, as determined by a high-throughput assay^12^.

At position −1 of HPV16E6 PBM, a glutamine forms a H-bond with a water molecule that is also H-bonded to the N524 of the PDZ domain of PTPN3, whereas the N531 of PTPN4 forms a H-bond with the D in position −1 in the PBM of the cellular partner GluN2A-16^11^. This asparagine is very well conserved in the orthologs of PTPN3 and PTPN4, while a serine or threonine is more commonly found at this position in the PDZome.

N524 and S538 (S545 for PTPN4) form also H-bonds with the E in position −3 of the HPV16E6 PBM (Figs 3B and 4A). S538 and N524 are strictly conserved in PTPN3 and PTPN4 orthologues, with only 2 and 1 exceptions respectively (Fig. 6); although in the PDZome, S or T residues are often located at this position 524, and in position 538, we often find S or T but also K, H, Q and R. E_−3_ is also stabilized by K526 in PTPN3. This K is conserved in PTPN3 and PTPN4 orthologues but only a few K are present at this position in the human PDZome and I, V, A, S and M are more often found. We previously reported that one single mutation (Q to E) at position −3 of the PBM of the envelope G protein of rabies virus (RABV) switches the fate of the infected cell from survival to apoptosis^32^. Indeed, the Q to E change increases the number and change the pattern of cellular partners for the PBM in the infected cells, enabling the attenuated RABV strain G protein to interact with PTPN4 inducing cell death. PTPN4-PDZ is able to discriminate between these peptides, displaying a substantially higher affinity for E rather than Q in position −3. Altogether, these observations and the fact that a glutamic acid is often found in position −3 in all PBMs interacting with PTPN3 and PTPN4^3,4,10,11,19,20,32,33^ suggest that interactions with this E in position −3 are a critical determinant of specificity of PDZ/PBM interaction in the NT5 group comprising PTPN3 and PTPN4 phosphatases.

Finally, the arginine in position −4 is linked by ionic bonds to D573 in PTPN3-PDZ (D580 in PTPN4-PDZ) complexed to HPV16E6 PBM. This interaction is most likely maintained in the complexes with viral HPV18E6 PBM, HBVc PBMs, and even with the p38γ PBM, which presents a lysine instead of an arginine. D573 is well conserved in the orthologous PTPN3 and PTPN4 with only 3 exceptions, yet it is not the most frequently found amino acid at this position in the human PDZome. It might also be a specificity determinant common to the PDZ of PTPN3 and PTPN4 phosphatases. These two closely-related non-receptor PTPs that compose the NT5 subfamily emerged in holozoa^34^ and duplicated in vertebrates with 54% of sequence identity, which rises to 71% for their PDZ domains. However, the expression pattern, substrates and interacting partners of PTPN3 and PTPN4 have a limited overlap.

## Methods

### Production and purification of recombinant proteins and PBM ligands

PTPN3-PDZ and PTPN3-PDZ_Next_ (Fig. 1) are encoded as an N-terminal gluthathione S-transferase (GST) tagged protein in a pDEST15 expression plasmid and a pGST//2 (derived from pGEX-4T-1; Amersham) expression plasmid respectively. A TEV cleavage site is introduced between the N-terminal tag and the protein sequence. The vectors were used to transform *E. coli* BL21 Star (DE3) star cells (Invitrogen, Carlsbad, CA, USA). Uniformly ^15^N-labeled, ^13^C, ^15^N-labeled and unlabeled PTPN3-PDZ and PTPN3-PDZ_Next_ constructs were expressed and purified as previously described^9^.

Briefly, harvested cells were resuspended in buffer A (50 mM Tris/HCl, pH 7.5, 150 mM NaCl), 2 mM β-mercaptoethanol and protease inhibitor cocktail (Roche), and then disrupted in a French press. The clarified supernatants were loaded onto a GST column (GSTrap HP, GE Healthcare) and washed with the same buffer. The GST tag was cleaved by overnight incubation at 4 °C by TEV protease (1% mol/mol) directly injected into the column. The eluted fractions containing the protein were pooled and loaded onto a size exclusion column (HiLoad Superdex 75 pg; GE) equilibrated with buffer A with 0.5 mM Tris(2-carboxyethyl)phosphine (TCEP). For crystallogenesis of PTPN3-PDZ_Next_, the same protocol was followed, replacing the Tris/HCl in buffer A by 20 mM HEPES pH 8 on the size exclusion chromatography step. Purified proteins were concentrated using centrifugal filter devices (Vivaspin, Sartorius). Protein concentration was estimated from its absorbance at 280 nm.

The peptides, p38γ PBM, HBVc PBM, HPV16E6 PBM and HPV18E6 PBM, were synthesized in solid phase using Fmoc strategy (Proteogenix) and resuspended in H_2_O.

### CD experiments

All CD measurements were acquired with an Aviv 215 spectropolarimeter. Far‐UV (195–240 nm) spectra were recorded at 25 °C on 8.4 μM PTPN3-PDZ samples in a cylindrical cell with a 0.2‐mm path‐length. Ellipticity was measured every 1 nm. The final spectrum of the protein sample was obtained by averaging three successive scans and subtracting the baseline spectrum of the buffer recorded under the same conditions. The CONTIN program was used for quantitative decomposition of the far‐UV CD spectrum^35^.

### NMR experiments

The NMR samples for the PTPN3-PDZ and PTPN3-PDZ_Next_ constructs were prepared in buffer A with 0.5 mM TCEP and D_2_O (5–10% vol:vol). All NMR experiments were performed on a 600-MHz Varian NMR System spectrometer equipped with a triple resonance ^1^H{^13^C/^15^N} cryoprobe.

The NMR titration experiments to measure PTPN3-PDZ·PBM peptide affinities and the NMR experiments for backbone assignment of PTPN3-PDZ in complex with HPV16E6 PBM were performed with the PTPN3-PDZ construct at 15 °C. Briefly, the unlabeled peptides (stock solutions ranging from 2.8 to 5.7 mM) at pH 7.5 were added stepwise in a sample initially containing 240–260 μL of ^15^N-labeled PTPN3-PDZ at a concentration of 95 or 149 μM. A series of ^1^H, ^15^N HSQC spectra was recorded for the different titration points. Chemical shift changes were calculated using the free PTPN3-PDZ signals as a reference. Chemical shift differences in the cross-peaks by titration were calculated using the relationship: Δ*δ* = ((Δ*δ*_HN_)^2^ + (0.15 × Δ*δ*_N_)^2^)^0.5^ where Δ*δ*_HN_ and Δ*δ*_N_ are changes in the ^1^H and ^15^N chemical shifts in ppm, respectively. The weighting factor of 0.15 was used to adjust the relative magnitudes of the amide nitrogen chemical shift range and the amide proton chemical shift range. Signals broaden in the moderate fast-exchange regime observed with PTPN3-PDZ and the PBM peptides, increasing the experimental errors on the chemical shift measurements used for the fitting of the Kd. The following of chemical shift changes during titrations and the fitting of curves were performed with the CcpNmr Analysis software^36^. A pool of 8 to 14 peaks with the best fit for each titration were kept to deduce the K_D_, and the errors are the standard deviations of all the K_D_ values fitted from the curves.

The sequence specific ^1^H^N^, ^15^N, ^13^C^α^, ^13^C^β^ and ^13^CO resonance assignments were performed using TROSY-based versions of the following experiments: 2D ^1^H, ^15^N HSQC and 3D HNCO, HNCA, HN(CO)CA, CBCA(CO)NH and HNCACB. The spectra were processed with NMRPipe^37^ and subsequently analysed in CcpNmr Analysis software^36^. The chemical shifts have been deposited in the BioMagResBank (BMRB) under accession number 27645.

A series of ^1^H, ^15^N HSQC spectra was recorded over a week at 20 °C for a sample of PTPN3-PDZ_Next_ at 170 μM to follow the stability of the construct.

### DSC experiments

DSC experiments were carried out on a Microcal VP-capillary DSC (Malvern). 18, 21, 94 and 28 μM of PTPN4-PDZ, free PTPN3-PDZ, free PTPN3-PDZ_Next_ and PTPN3-PDZ complexed with peptides respectively were scanned from 10 °C to 90–100 °C with a scan rate of 200 °C/h. Large excess of peptides were used: about 600 μM for p38γ PBM, about 300 μM for HBVc PBM and HPV16E6 PBM and about 200 μM for HPV18E6 PBM. The buffer A was repeatedly scanned to ensure a stable buffer baseline. The buffer baseline was subtracted from the protein thermogram. Subsequent to normalization of the data by the protein concentration, a non-linear curve fitting algorithm was employed to obtain the Tm of the transition. All experiments were repeated three times.

### AUC experiments

Sedimentation velocity experiments were carried out at 20 °C using a Beckman Coulter XL-I centrifuge equipped with a AN60-Ti rotor. Various protein and protein–peptide complex concentrations (protein concentrations ranging from 12.5 to 70 μM) samples were centrifuged for 17 h at 42000 rpm. Data were analyzed with SEDFIT 15.1 using a continuous size distribution c(S) model. The partial specific volume, the viscosity and the density of the samples were calculated with SEDNTERP. The processed data were used to obtain values of sedimentation coefficients at null concentration in our experimental conditions (S0) and to get the standard sedimentation coefficients in water at 20 °C (S_0,w,20_) (Table 2).

### Crystallisation, data collection, and structure determination

The HPV16E6 PBM peptide used for co-crystallization was added in excess to form >95% of the complex with the protein. The PDZ domain-peptide complex for crystallization was generated by mixing PTPN3-PDZ_Next_ and the peptide at a ratio of 1:2. Initial screening of crystallization conditions was carried out by the vapor diffusion method using a MosquitoTM nanoliter-dispensing system (TTP Labtech). Sitting drops were set up using 400 nL of a 1:1 mixture of each sample protein and crystallization solutions (672 different commercially available conditions) equilibrated against a 150 μL reservoir in multiwell plates (Greiner Bio-One). The crystallization plates were stored at 4 °C in a RockImager1000^®^ (Formulatrix) automated imaging system to monitor crystal growth. The best crystals were obtained by mixing 200 nL of PTPN3-PDZ_Next_· HPV16E6 PBM complex solution (concentration of the PDZ domain at 4.8 mg/mL) in 20 mM HEPES pH 8, 150 mM NaCl, 0.5 mM TCEP mixed with 200 nL of reservoir solution containing 20% w/v PEG 3350, 0.2 mM KI at pH 7. Crystals were then flash-cooled in liquid nitrogen using Paratone-paraffin 50%(V/V)/50%V/V) oil as the cryoprotectant.

X-ray diffraction data were collected at a wavelength of 0.979 Å on the beamline PROXIMA-2A at Synchrotron SOLEIL (St. Aubin, France). The data were processed with XDS^38^ and Xdsme^39^, and other programs from the CCP4 suite^40^. The structures were solved by molecular replacement with PHASER^41^ using the search atomic model of PTPN4-PDZ (PDB ID 5EZ0). The locations of the bound peptides were determined from a *F*_*o*_–*F*_*c*_ difference electron density maps. Models were rebuilt using COOT^42^, and refinement was done with phenix.refine of the PHENIX suite^43^. The overall assessment of model quality was performed using MolProbity^44^. The crystal parameters, data collection statistics, and final refinement statistics are shown in Table 3. All structural figures were generated with the PyMOL Molecular Graphics System, Version 1.7 (Schrödinger).

### Sequence alignment

Sequence of PTPN3 (accession number NP_002820.3) was used as query on the InterEvolAlign server^45^ to retrieve one single homolog per species assessed as probable ortholog through a reciprocal blast search procedure against the non-redundant database. Retrieved full-length sequences were re-aligned using MAFFT^46^ and displayed using Jalview^47^.

## Supplementary information

Supplementary Figure S1

## Data Availability

All data generated or analysed during this study are included in this published article.
