# Supplementary material for: Structural and functional characterization of the PDZ domain of the human phosphatase PTPN3 and its interaction with the human papillomavirus E6 oncoprotein
Source: Sci Rep. 2019 May 15;9:7438. doi: 10.1038/s41598-019-43932-x (PMC6520365; doi:10.1038/s41598-019-43932-x)
Supplement: Supplementary file 1 — Supplementary Figure S1 [file 41598_2019_43932_MOESM1_ESM.pdf]

Structural and functional characterization of the PDZ domain of the Human Phosphatase PTPN3 and its interaction with the human papillomavirus E6 oncoprotein

Mariano Genera[1,2], Damien Samson[3], Bertrand Raynal[4], Ahmed Haouz[5], Bruno Baron[4], Catherine Simenel[3], Raphael Guerois[6], Nicolas Wolff[1] and Célia Caillet-Saguy[1,*]

[1] Récepteurs-Canaux, Institut Pasteur, UMR 3571, CNRS, F-75724 Paris, France

[2] Sorbonne Université, Complexité du Vivant, F-75005 Paris, France

[3] RMN des biomolécules, Institut Pasteur, UMR 3528, CNRS, F-75724 Paris, France.

[4] Plate-forme de Biophysique Moléculaire, Institut Pasteur, UMR 3528, CNRS F-75724 Paris, France

[5] Plate-forme de Cristallographie, Institut Pasteur UMR 3528, CNRS , F-75724 Paris, France

[6] Institut de Biologie Intégrative de la Cellule (I2BC), CEA, CNRS, Université Paris-Sud, Université Paris-Saclay, 91190 Gif-sur-Yvette Cedex, France

* Correspondence should be addressed to Célia Caillet-Saguy (email: celia.caillet-saguy@pasteur.fr)

Supplementary Figure S1

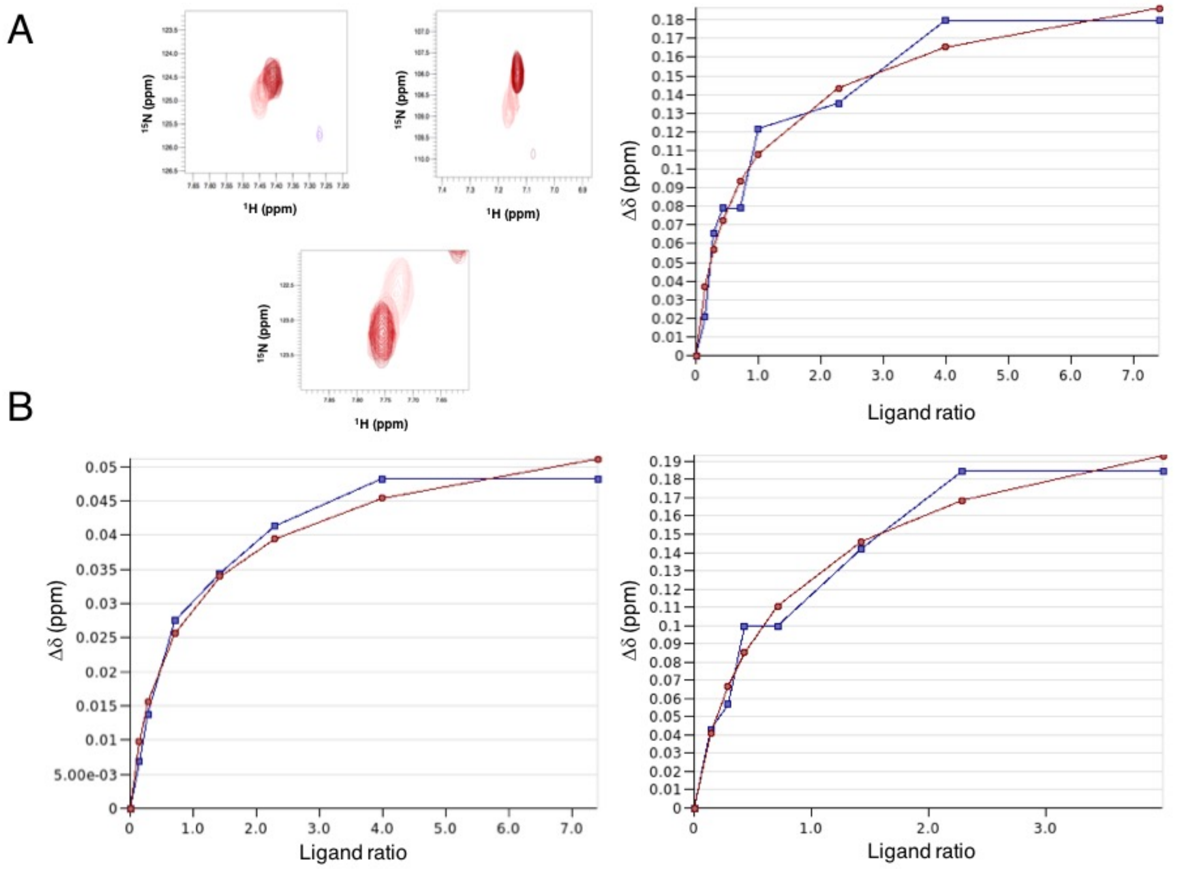

Supplementary Figure S1: K_D determination using NMR 2D-HSQC-type experiment. (A) Chemical shift changes of several peaks in the absence and presence of different amounts of HPV16 E6 PBM peptide; (B) Plots of the chemical shift changes upon peptide binding against [peptide]/[protein] ratio extracted from Ccpnmr analysis. The fitted and experimental values are represented as red and blue dots respectively and experimental dots are related by a blue line while the fitted dots are related by a red line reflecting the fitted curve.
